# A Lysozyme-Derived Peptide Induces GLP‑1 Secretion in Mouse Jejunal Organoids and Modulates Glycemia in Rats

**DOI:** 10.1021/acs.jafc.5c05148

**Published:** 2025-06-18

**Authors:** Santiaga María Vivanco-Maroto, María-Carmen López de las Hazas, Esperanza Herradón, Rocío Girón, Beatriz Miralles, Nancy Paniagua, Visitación López-Miranda, Alberto Dávalos, Isidra Recio

**Affiliations:** † 16379 Instituto de Investigación en Ciencias de la Alimentación, CIAL (CSIC-UAM, CEI UAM+CSIC), Nicolás Cabrera, 9, Madrid 28049, Spain; ‡ Laboratory of Epigenetics of Lipid Metabolism, Madrid Institute for Advanced Studies Food (IMDEA Food), CEI UAM + CSIC, Carretera de Canto Blanco, 8, Madrid 28049, Spain; § Área de Farmacología, Nutrición y Bromatología, Dpto. C.C. Básicas de la Salud, Facultad de Ciencias de la Salud, Unidad Asociada I+D+i al Instituto de Química Médica (CSIC), Universidad Rey Juan Carlos, Alcorcón 28922, Spain; ∥ High Performance Research Group in Experimental Pharmacology (PHARMAKOM), Universidad Rey Juan Carlos (URJC), Madrid 28922, Spain; ⊥ Consorcio CIBER de la Fisiopatología de la Obesidad y Nutrición (CIBERObn), Instituto de Salud Carlos III (ISCIII), Madrid 28029, Spain

**Keywords:** GLP-1, mouse jejunal organoids, egg white peptides, type 2 diabetes, enteroendocrine cells

## Abstract

Protein digestion products promote the release of incretins, such as GLP-1, which regulate glucose homeostasis. Our previous studies demonstrated that the egg white peptide fraction stimulates GLP-1 secretion in STC-1 cells. Here, the GLP-1 secretion induced by the lysozyme-derived peptide ^123^WIRGCRL^129^ and six alanine-substituted analogues was evaluated in mouse jejunal organoids, alongside egg white digest and the amino acid phenylalanine (Phe). Phe induced a faster GLP-1 response, but the peptide ^123^WIRGCRL^129^ elicited a similar GLP-1 response, although tested at a 20-fold lower concentration. The GLP-1 release in organoids elicited by the peptide was 57.8 ± 5 pM, while Phe reached 43.7 ± 1 pM at 60 min. In STC-1 cells, the peptide induced 667.7 ± 24.2 pM of GLP-1 compared to 416.6 ± 40.1 pM induced by Phe. Results obtained in STC-1 suggested the involvement of ERK- and AMPK-mediated pathways in the GLP-1 secretion induced by the peptide. Oral glucose tolerance tests in Wistar rats after oral administration of ^123^WIRGCRL^129^ showed a reduction in glucose levels, while no changes were observed in the group receiving the amino acid mixture at equimolar concentration. These findings suggest the potential therapeutic application of some food peptides against type 2 diabetes.

## Graphical abstract

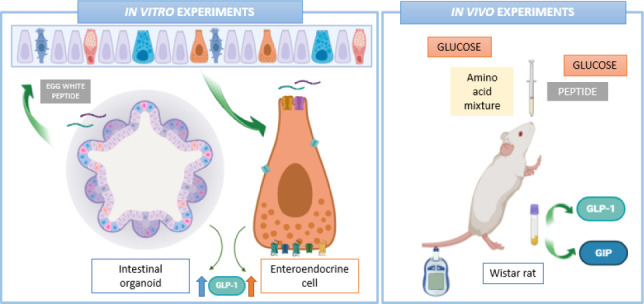

## 1 Introduction

The intestinal epithelium is the interface between the intestinal environment and food, and it coordinates the processes of digestion and nutrient absorption. Different types of cells constitute this epithelium, including enteroendocrine cells. Although they constitute only 1% of the cellular composition, they are capable of acting as true nutrient sensors at the intestinal level and release various hormones in response to food intake.

Dietary protein intake is a potent stimulus inducing the secretion of these hormones, including glucagon-like peptide-1 (GLP-1). GLP-1 is an incretin responsible for controlling food intake and glucose homeostasis, including blood glucose levels. Different food proteins, protein hydrolysates, and free amino acids have been demonstrated to induce GLP-1 release in human studies.^1^ Intraduodenal infusion of a whey protein hydrolysate triggered GLP-1 release and reduced glucose concentration in both lean and obese subjects.^2^ The free amino acid glutamine enhanced postprandial glucagon and insulin response in healthy and diabetic patients.^3,4^

Several authors have suggested that the peptide fraction and, specifically, oligopeptides, rather than small peptides or free amino acids, are the main GLP-1 inducers. Cordier-Bussat et al. (1998) demonstrated that egg and meat hydrolysates could increase GLP-1 levels produced in enteroendocrine cells and, in *ex vivo* rat intestinal models, the effect was attributed to oligopeptides.^5^ Later, other authors, using the enteroendocrine cell line STC-1, have related the GLP-1-inducing effect to peptides of molecular mass between 500 and 1500 kDa,^6^ and it was shown that peptides present a greater inducing effect than amino acids on GLP-1 secretion.^7,8^ A small number of peptide sequences from bovine hemoglobin,^6^ egg white,^8^ tilapia,^9^ and β-casein^10^ have been demonstrated to be capable of stimulating GLP-1 release, and the ability of some peptides to regulate glucose levels has been demonstrated in animal models, such as the β-casein peptide ^70^LPQNIPPL^77^ identified in Gouda cheese.^11^ However, the peptide structural features required to induce GLP-1 secretion are not fully understood. In a previous screening of peptides from milk and egg white proteins in STC-1 cells, three sequences from egg white lysozyme, including the ^123^WIRGCRL^129^ peptide, were identified as potent inducers of GLP-1 secretion in the STC-1 cell line. These results demonstrated that the amino acid sequence was crucial to the inducing effect, and amino acid deletions induced the loss of the secretagogue effect.^12^ However, whether this effect is observed in a more complex model, different than a cell line, or whether a physiological effect is observed *in vivo* is unknown.

Enteroendocrine cell lines, such as STC-1, GLUTag, or NCI-H716, are valuable *in vitro* models to investigate intestinal hormone secretion and the involved mechanisms, but these lines present certain limitations. In particular, STC-1 cells express low levels of the peptide transporter 1 (PEPT-1) and of some K^+^ channel subunits, which are important in the functioning of sodium-coupled glucose transporters (SGLTs).^13,14^ Thus, intestinal organoids have emerged as a more physiologically relevant approach, as they are a stem cell-derived 3D culture model that simulates the phenotypic structure, cellular composition, and partial function of the small intestine.^15^

The aim of this study is to investigate the GLP-1 secretion induced by a lysozyme-derived peptide, previously identified as a potent secretagoguein STC-1 cells, in mouse jejunal organoids. In order to explore the sequence requirements, a peptide library was built by substituting each amino acid with alanine. The pathways involved in the GLP-1 secretagogue effect of the lysozyme-derived peptide were explored in both STC-1 and jejunal organoids, and the ability of this sequence to regulate glucose levels was evaluated in an oral glucose tolerance test in Wistar rats.

## 2 Materials and Methods

### 2.1 Chemicals and Reagents

Egg white was manually separated from ecological fresh eggs obtained in a local supermarket and was digested according to the INFOGEST *in vitro* protocol.^16^ The freeze-dried supernatant of the heat-treated digest, after centrifugation at 5000 × *g* for 20 min, is termed EWD. Synthetic lysozyme-derived peptide ^123^WIRGCRL^129^ and its modifications AIRGCRL, WARGCRL, WIAGCRL, WIRACRL, WIRGARL, WIRGCAL, and WIRGCRA were purchased from CSBIO Ltd. (Shanghai, China). All peptides were supplied as lyophilized powders, and their purity (>90%) was verified by elemental analysis, reverse-phase high-performance liquid chromatography, and tandem mass spectrometry. The amino acids included in the amino acid mixture were obtained from Sigma-Aldrich (Darmstadt, Germany) with a purity ≥97% and were suitable for use in cell culture. DMEM/F12 and PSA were sourced from Lonza (Basel, Switzerland). Matrigel was purchased from Corning (NY, USA). IntestiCult Organoid Growth Medium was obtained from STEMCELL Technologies (Vancouver, Canada). Phenylalanine (Phe), HEPES, glucose, NaCl, KCl, CaCl_2_, MgCl_2_, BSA, and formic acid were supplied by Sigma-Aldrich (Darmstadt, Germany). QIAzol, miRNeasy Mini Kit, Script II RT Kit, and miScript SYBR Green PCR Kits were procured from Qiagen (Hilden, Germany). STC-1 cells, DMEM, and fetal bovine serum were obtained from ATCC (Manassas, USA). GLP-1 ELISA and GIP ELISA kits were supplied by Merck KGaA (Darmstadt, Germany). Antibodies were acquired from Cell Signaling Technology (Danvers, MA, USA).

### 2.2 Mouse Intestinal Organoid Culture

Mouse intestinal organoids (enteroids) were derived from adult intestinal stem cells (ISCs). Intestinal crypts were isolated from the jejunum of male and female wild-type mice following the protocol provided by STEMCELL Technologies. Crypts were cultured at 37 °C until organoids were fully developed (5–7 days).^17^ Intestinal organoids were suspended in 10 mL of cold DMEM/F12 (Lonza, Basel, Switzerland), and the crypts were suspended in a mixture (50:50) of Matrigel (Corning, NY, USA) and complete IntestiCult Organoid Growth Medium (STEMCELL Technologies, Vancouver, Canada). This mix was gently placed in the center of each well of a prewarmed 24-well plate until the Matrigel solidified and formed a dome. IntestiCult Organoid Growth Medium (STEMCELL Technologies, Vancouver, Canada) was added, and crypts were cultured at 37 °C and 5% CO_2_ until organoids were developed. After 7 days, the mouse intestinal organoids were passed following the STEMCELL Technologies protocol. The experiments were performed from passage numbers 1 to 6.

#### 2.2.1 Hormonal Secretion Studies in Organoids

On the day of the experiment, the medium was discarded, and the Matrigel was removed with cold PBS. Next, the organoids were centrifuged for 3 min at 300 × *g*, the medium was discarded, and after three washes, the organoids were resuspended in saline buffer (20 mM HEPES 1 M, 1 mM glucose, 140 mM NaCl, 4.5 mM KCl, 1.2 mM CaCl_2_, 1.2 mM MgCl_2_, 0.1% BSA, pH 7.4). The organoids were incubated for 40 min at 37 °C in this buffer. These organoids were resuspended in a new saline buffer and transferred to 96-well plates at a 60 organoids per well-ratio. The samples (EWD at 2 mg/mL, Phe at 20 mM, and synthetic peptide at 1 mM) were diluted in saline buffer, added to the well, and assayed in triplicate. This concentration was selected as it had been shown to be viable in STC-1 cells. After 10, 30, and 60 min, the plate was centrifuged for 3 min at 300 × *g*, supernatants were collected, organoids were lysed with QIAzol (Qiagen, Hilden, Germany), and samples were stored at −80 °C for further analysis. Preliminary experiments showed that, upon exposure to a casein hydrolysate, GLP-1 release of cultured organoids in domes produced a consistent, although lower, response than in free form (results not shown). Working in suspension form allowed a better adjustment of the number of organoids per well and a higher recovery of GLP-1.

### 2.3 Cell Culture

STC-1 cells supplied by ATCC (ATCC CRL-3254, Manassas, VA, USA) were cultured in Dulbecco’s Modified Eagle’s Medium (ATCC, Manassas, VA, USA) supplemented with 100 U/mL penicillin, 100 mg/L streptomycin, 0.25 μg/mL amphotericin (Lonza Group, Basel, Switzerland), and 10% fetal bovine serum (ATCC, Manassas, VA, USA). Cells were incubated at 37 °C in a 5% CO_2_ humidified atmosphere, and once they reached 80% confluence, they were trypsinized and seeded according to each cell study. The experiments were performed from passage numbers 31–35.

#### 2.3.1 Hormonal Secretion Studies in STC-1 Cells

Cells were cultured at 37 °C, 5% CO_2_, and a humidified atmosphere for 48 h into 24-well plates at a density of 3 × 10^5^ cells per well. Before the assay, the medium was removed, and the cells were washed three times with HEPES buffer (20 mM HEPES 1 M, 10 mM glucose, 140 mM NaCl, 4.5 mM KCl, 1.2 mM CaCl_2_, 1.2 mM MgCl_2_, pH 7.4) and incubated 1 h prior to addition of samples or HEPES buffer (control). The samples (EWD at 2 mg/mL, Phe at 20 mM, and synthetic peptide at 1 mM) were tested in triplicate. After 10, 30, and 60 min, supernatants were collected as described in ref 18, cells were lysed with QIAzol (Qiagen, Hilden, Germany), and samples were stored at −80 °C.

### 2.4 GLP-1 Secretion

After hormonal secretion studies, GLP-1_7–36NH2_ was measured in the supernatants of cells or organoids in duplicate with the commercial immunoassay Glucagon-Like Peptide-1 Active ELISA (Merck KGaA, Darmstadt, Germany), according to the manufacturer’s instructions. The samples of this study did not show cross-reactivity with the GLP-1-specific antibodies (7–36) and (9–36) used in this immunoassay.

### 2.5 Gene Expression

For RNA isolation, frozen STC-1 cells or organoids were homogenized in QIAzol, and total RNA was isolated using the miRNeasy Mini Kit (Qiagen, Hilden, Germany). Total RNA was quantified in a NanoDrop 2000 spectrophotometer (Thermo Scientific, MA, USA). According to the instructions, total RNA was reverse transcribed to cDNA using a Script II RT Kit (Qiagen, Hilden, Germany). RNA expression levels were determined in duplicate by quantitative real-time PCR (qPCR) on a 7900HT Real-Time PCR System (Applied Biosystems), using miScript SYBR Green PCR Kits (Qiagen, Hilden, Germany).

Amplification was initiated at 50 °C for 2 min and at 95 °C for 10 min, followed by 40 cycles of 95 °C for 15 s and 60 °C for 1 min. The following specific oligonucleotides were used: for cholecystokinin (*CCK*) forward (F) 5′-CCAATTTTTCCTGCCCGCAT-3′ and reverse (R) 5′-AGAAGGAGCAGTCAAGCCAAA-3′; for *GLP-1* (F) 5′-AGAGACATGCTGAAGGGACC-3′ and (R) 5′-CTTTCACCAGCCACGCAATG-3′; for peptide YY (*PYY*) (F) 5′-GGACGCCTACCCTGCCAAACCA-3′ and (R) 5′-AGTGCCCTCTTCTTAAACCAAACA-3′; and for reference gene β-*actin* (F) 5′-AGCTGCGTTTTACACCCTTT-3′ and (R) 5′-AAGCCATGCCAATGTTGTCT-3′. The relative expression levels of the target gene were calculated using the comparative critical threshold method (2^–ΔΔCt^), by normalizing the data to the expression of β-actin.

### 2.6 Peptide Identification in Organoid Supernatants

High-performance liquid chromatography–tandem mass spectrometry (HPLC–MS/MS) analyses were performed on an Agilent 1100 series HPLC separation system (Agilent, Palo Alto, CA, USA) coupled online with an Esquire 3000 linear ion trap mass spectrometer (Bruker Daltonics GmbH, Bremen, Germany). The analytical column used was a Gemini 3 μm NX-C18, 150 × 2 mm (Phenomenex, Madrid, Spain). Elution with a linear gradient (0 to 45 in 60 min) of solvent B (acetonitrile:formic acid, 100:0.1, v/v) in solvent A (water:formic acid, 100:0.1, v/v) was used. Supernatants were diluted 1:5 in eluent A. Mass spectra were acquired in the range of 100–1200 *m*/*z* with the target mass fixed at 750. Data were processed with the software programs DataAnalysis 4.0, BioTools 3.2, and SequenceEditor (Bruker Daltonics, Bremen, Germany). The percentage of each sequence was calculated relative to the total area in each sample.

### 2.7 Immunoblotting

Organoids and STC-1 cells were exposed to samples or assay buffer for 5, 15, and 30 min; then, cells were lysed with RIPA buffer, and ERK1/2, p-ERK1/2, AMPK, p-AMPK, p-IRS-1, IRS-1, glyceraldehyde-3-phosphate dehydrogenase (GAPDH), and heat shock protein (HSP90) contents were examined using immunoblotting. Protein samples were lysed, separated on 10% SDS-polyacrylamide gels, and the separated proteins were electroblotted from the gel onto nitrocellulose membranes (0.22 μM, Millipore, MA, USA) and blocked with skim milk for 30 min at room temperature. Membranes were incubated with primary antibodies overnight at 4 °C. After incubation, membranes were exposed to secondary antibodies for 45 min at room temperature. Blots were rinsed three times with Tris-buffered saline with Tween 20, pH 8.0, after each incubation step and visualized using an Odyssey infrared imaging system (LI-COR, Lincoln, NE, USA).

### 2.8 Animal Care

Experiments were performed on 8-week-old Wistar male rats weighing between 280 and 310 g, purchased from Envigo (Gannat, France), according to the European Union guidelines for the use of laboratory animals and in compliance with the ethical guidelines for studies on experimental animals. This study has been approved by the Universidad Rey Juan Carlos Animal Committee (PROEX 017.1/22). The rats were housed in a temperature- and humidity-controlled room, under a 12 h light/dark cycle, and fed *ad libitum* with a standard rodent diet (Altromin Spezialfutter GmbH & Co. KG, Germany). The experiments were performed after an acclimation period.

### 2.9 Glycemic Response in Wistar Rats under Oral Glucose Tolerance Test (OGTT)

After overnight fasting, the glucose levels were tested in all animals on the day of the experiment. 45 min before the glucose challenge, rats were orally administered water, peptide, or amino acid mixture at a concentration of 0.1 mM/kg of body weight. Next, glucose levels in the blood were measured, and water (in the case of the basal group) or 3 g/kg of glucose (peptide, amino acid mixture, and overload groups) was administered. Each group included six animals. All intragastric administrations contained 0.5 mL of volume, and the glucose levels in the blood were measured at 15, 30, 60, or 120 min after that with an Accu-Chek glucometer (Roche, Basel, Switzerland).

### 2.10 GLP-1 and GIP Levels in Plasma Samples

Rats were fasted overnight before the experiment. The glucose levels were tested in all animals on the day of the experiment. 45 min before the glucose challenge, rats were orally administered water, peptide, or amino acid mixture at a concentration of 0.1 mM/kg of body weight. All intragastric administrations contained 0.5 mL of volume. Next, glucose levels in the blood were measured, and water (in the case of the basal group) or 3 g/kg of glucose (peptide, amino acid mixture, and overload groups) was administered. 10 min after this administration, the animals were anesthetized (sodium pentobarbital 50 mg/kg by intraperitoneal injection), and 15 min later, following the water/glucose overload, blood glucose levels were, again, analyzed. Just after this, rats were sacrificed (under anesthesia), blood was collected in tubes with EDTA and protease inhibitors (Roche, Basel, Switzerland), plasma was obtained, and gastric inhibitory polypeptide (GIP) (Merck KGaA, Darmstadt, Germany) and GLP-1 levels were measured according to the manufacturer’s instructions.

### 2.11 Statistical Analysis

Hormonal secretion, gene expression, protein expression, and OGTT were compared using one-way ANOVA with Tukey’s post hoc test for pairwise comparisons. The results were considered significant if *p* < 0.05. GraphPad Prism version 8 for Windows (GraphPad Software, San Diego, CA, USA) was used for graphics and calculations.

## 3 Results and Discussion

### 3.1 Kinetics of GLP-1 Secretion in Response to Lysozyme-Derived Peptide ^123^WIRGCRL^129^, EW, and Phe in Organoids and STC-1 Cells

Hormonal expression and GLP-1 release induced by the lysozyme-derived peptide ^123^WIRGCRL^129^ were evaluated in jejunal organoids over 1 h using EWD and Phe as complex (whole digest) and simple positive controls, respectively. A simulated egg white gastrointestinal digest had previously shown a potent GLP-1 and CCK secretagogue effect in an STC-1 culture.^8^ The amino acid Phe has been shown to specifically trigger GLP-1 and CCK responses, as demonstrated in rat intestinal loops^19^ and pig intestinal tissue.^20^ GLP-1 levels after incubation with these three products for 10, 30, and 60 min are shown in Figure 1A. Whereas a significant GLP-1 release was produced by the peptide after 60 min, a faster GLP-1 induction was observed in the case of Phe (10 min), while GLP-1 was induced at all time points (significance reached at 10 and 60 min) in the presence of EWD, a sample that comprises both free amino acids (28% of the total nitrogen content) and peptides (72% of the total nitrogen content). Since GLP-1 accumulation was measured in the supernatant, similar levels to those observed at 10 min would be expected for Phe at 30 and 60 min. The lower GLP-1 levels at these time points may be attributed to the degradation of GLP-1 over time by the DPP-IV enzyme, which is present in the organoids. It is worth mentioning that the peptide elicited a GLP-1 response of the same order of magnitude than that observed for Phe, although it was tested at a 20 times lower concentration (peptide at 1 mM vs Phe at 20 mM). The relative *proglucagon* gene expression supported the strengthened effect of peptide ^123^WIRGCRL^129^ at 60 min, reaching a 2.4-fold change, and the rapid stimulus of Phe (1.8-fold change at 10 min) (Figure 1B–D). Although mouse jejunal organoids have been used for assessing GLP-1 responses to glucose, dipeptides,^21^ or noncaloric sweeteners,^22^ this is, to the best of our knowledge, the first study determining induced GLP-1 upon exposure of jejunal organoids to dietary egg-derived proteins and peptides. *Cck* and *pyy* mRNA levels were also evaluated (Figure S1). As observed for *proglucagon,* the peptide showed induced expression of *CCK* at 60 min, in contrast to the earlier induction of Phe, from 30 min, with maximum fold changes of 2.3 and 2.8. In the case of *PYY*, an effect of both the peptide and Phe was observed at 10 min, with Phe keeping the increased expression at 30 min and the peptide at 60 min. EWD produced no significant effect on the expression of any of these genes, as had been observed for *proglucagon*. The wide range of possible gene regulators in this digest might lie behind the varied transcription values observed. Recently, brewer’s spent yeast peptide fractions were shown to increase the expression of intestinal epithelial genes related to diabetes in mouse jejunal organoids^23^ where the induced expression of the incretins GLP-1 and GIP in the 1.5-fold change level was observed after the incubation with a dialysate from the gastrointestinal digest for 24 h.

**Figure 1:**
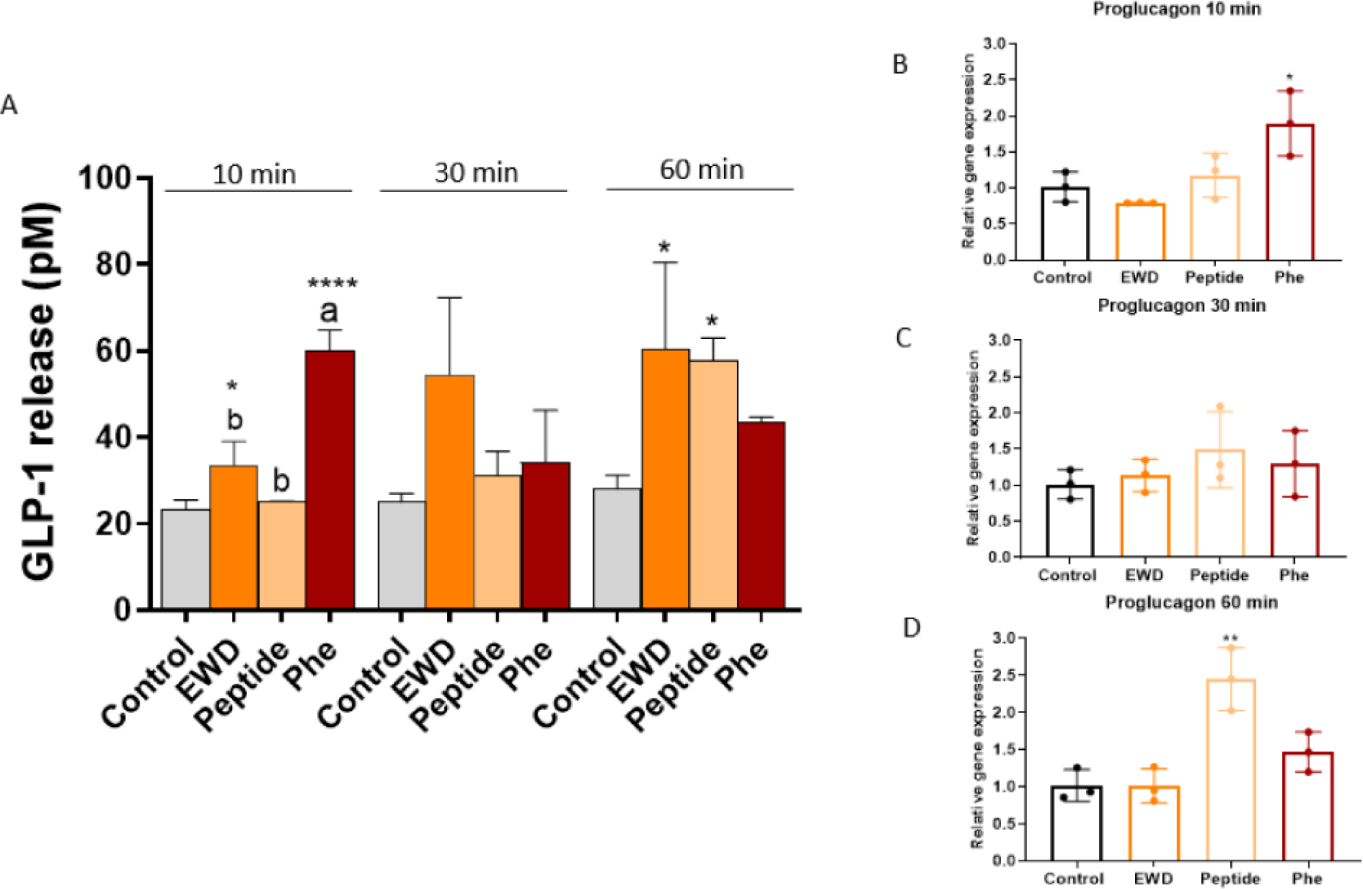
GLP-1 release at 10, 30, and 60 min after exposure to EWD, ^123^WIRGCRL^129^ (peptide), or Phe in mouse intestinal organoids (A) and relative proglucagon gene expression at 10 min (B), 30 min (C), and 60 min (D). EWD was assayed at 2 mg/mL, Phe at 20 mM, and synthetic peptide at 1 mM. Experiments were performed in triplicate, followed by technical duplicates. Error bars indicate SEM (*n* = 3). Statistical significance compared with control in GLP-1 release (one-way ANOVA with Tukey’s post hoc test) is indicated by *p < 0.05, **p < 0.01, ***p < 0.001, and ****p < 0.0001. Statistical significance (*p* < 0.05) in the comparison between different samples at the same time is indicated by different letters (a, b). Unpaired *t* test was used in gene expression and statistical significance compared with control is indicated by *p < 0.05, **p < 0.01, ***p < 0.001, and ****p < 0.0001.

STC-1 cells were used to build on the above observations. It has to be noted that this cell model produced quantitatively higher hormone levels, as it consists only of enteroendocrine cells. However, this cell line does not express the wide array of transporters and peptidases present in the stem cell-derived organoids. Figure 2A reveals that EWD produced a strong hormone release from 10 min, while levels of GLP-1 only reached significance after incubation with the peptide or Phe for 60 min. At this time, the amino acid triggered the lowest GLP-1 levels as compared to the other products, and the peptide showed an intermediate value. While, in organoids, induced secretion was observed in the presence of Phe at 10 min, in STC-1 cells, Phe induced GLP-1 only after 60 min and was less intense than the peptide or EW. A possible explanation comes from the fact that, although the STC-1 line expresses some amino acid-sensitive receptors such as G protein-coupled receptor family C group 6 member A (GPRC6A) and taste receptor type 1 member 3 (T1R3), it lacks others, such as T1R1, as previously was suggested.^24^ *Proglucagon* gene expression was studied at the same time points (Figure 2B–D), but the relative gene expression after the incubation with these samples was not statistically higher than the control at any of the studied time points. In the case of *CCK* and *PYY* gene expression, an outstanding effect of Phe could be observed after 60 min, where 4-fold and 2-fold gene overexpression was observed for *CCK* and *PYY*, respectively (Figure S2). No other changes in the expression level of these genes were observed at the studied times. In summary, peptide ^123^WIRGCRL^129^ is able to trigger GLP-1 release in both organoids and STC-1 cells in a time-dependent manner, with the effect becoming significant after 60 min. This stimulation is accompanied by a simultaneous overexpression of *proglucagon* and a concomitant increase in the *CCK* expression.

**Figure 2:**
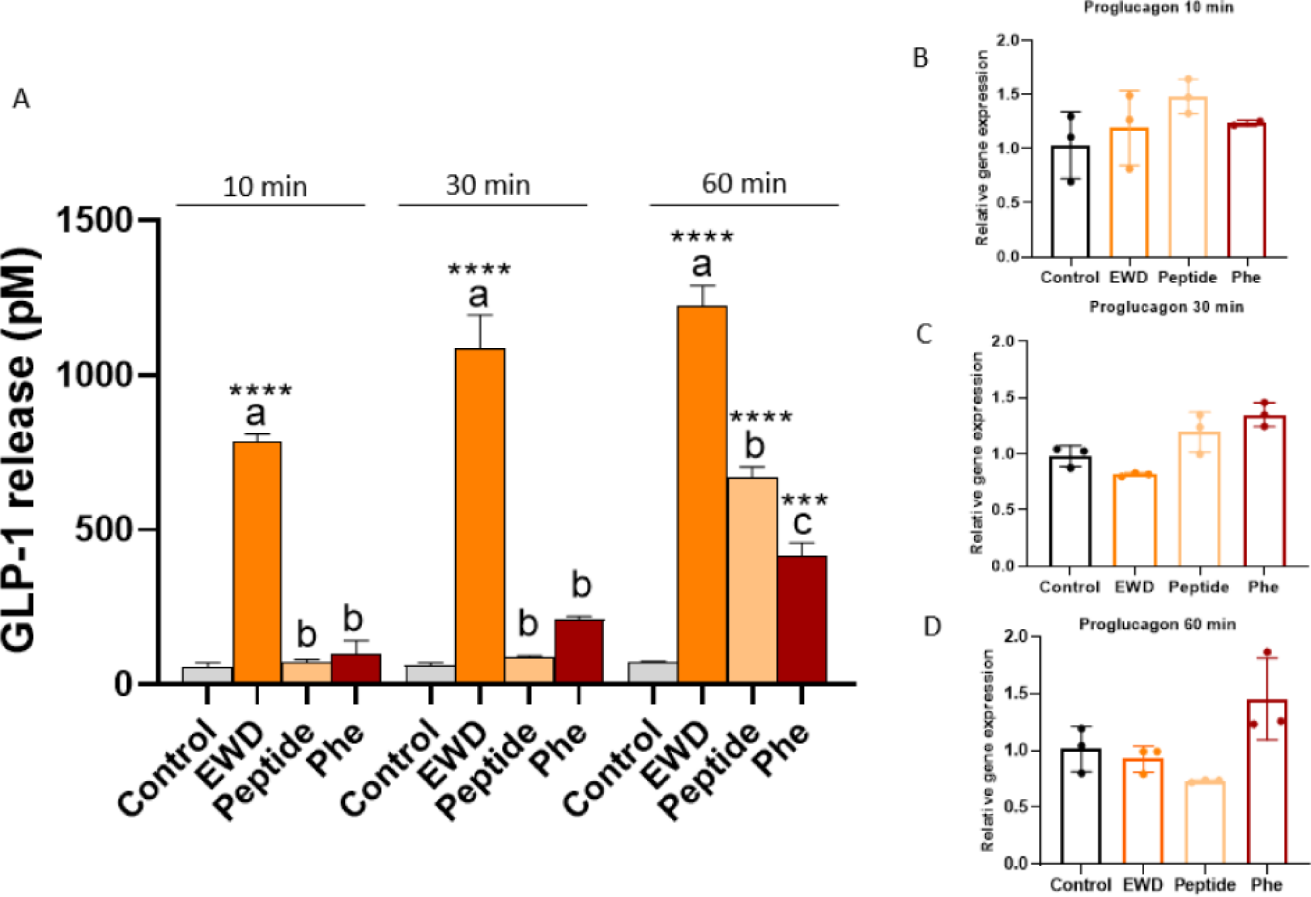
GLP-1 release at 10, 30, and 60 min after exposure to EWD, ^123^WIRGCRL^129^ (peptide), or Phe to STC-1 cells (A) and relative proglucagon gene expression at 10 min (B), 30 min (C), and 60 min (D). EWD was assayed at 2 mg/mL, Phe at 20 mM, and synthetic peptide at 1 mM. Experiments were performed in triplicate, followed by technical duplicates. Error bars indicate SEM (*n* = 3). Statistical significance compared with control (one-way ANOVA with Tukey’s post hoc test) is indicated by *p < 0.05, **p < 0.01, ***p < 0.001, and ****p < 0.0001. Statistical significance (*p* < 0.05) in the comparison between different samples at the same dose is indicated by different letters (a, b, c). Unpaired *t* test was used in gene expression and statistical significance compared with control is indicated by *p < 0.05, **p < 0.01, ***p < 0.001, and ****p < 0.0001.

Our results show that the GLP-1 response in a model that preserves the main phenotypic features and functional characteristics of the intestine, as occurs in mouse jejunal organoids, is slightly different than that found in STC-1 cells, especially for free amino acids like Phe, probably due to the limited expression of some amino acid receptors in the STC-1 cell line.

### 3.2 Sequence Requirements of ^123^WIRGCRL^129^ to Exert the GLP-1 Secretagogue Effect

In order to perform a comprehensive analysis of the sequence requirements of ^123^WIRGCRL^129^ to exert its GLP-1 secretagogue effect, a peptide library based on the parent peptide by substitution of each amino acid by alanine was synthesized and tested in organoids (Figure 3). The sequence with the substitution of ^128^R for alanine was not tested due to its poor solubility. Substitution of ^123^W, ^126^G, and ^129^L by alanine increased the GLP-1 levels compared to the original sequence, while the other substitutions did not modify the capacity to induce GLP-1 release. None of the sequences decreased GLP-1 secretion levels with regard to the ^123^WIRGCRL^129^ peptide. It is important to mention that all sequences have a high hydrophobicity (higher than 16.75) and are similar to each other, and some authors have described that peptides with hydrophobicity scores between 13 and 27 presented a strong secretory effect on GLP-1.^25^ Our previous study in STC-1 cells revealed that removal of the N-terminal ^123^W significantly reduced the GLP-1 response, and further elimination of ^124^I abolished the effects of this sequence.^12^ This shows the need for a minimum number of amino acids to exert the GLP-1 secretagogue effect, probably necessary to interact with the responsible receptors for triggering GLP-1 secretion. Recently, the role of the dipeptide WY, among many di- or tripeptides systematically studied, has been highlighted due to its high potential to stimulate GLP-1 secretion in the murine enteroendocrine GLUTag cells. The capability of GLP-1-secreting cells to specifically recognize the structure of the amino acid chains was confirmed, as the reverse sequence or free amino acids elicited no effect.^26^

**Figure 3:**
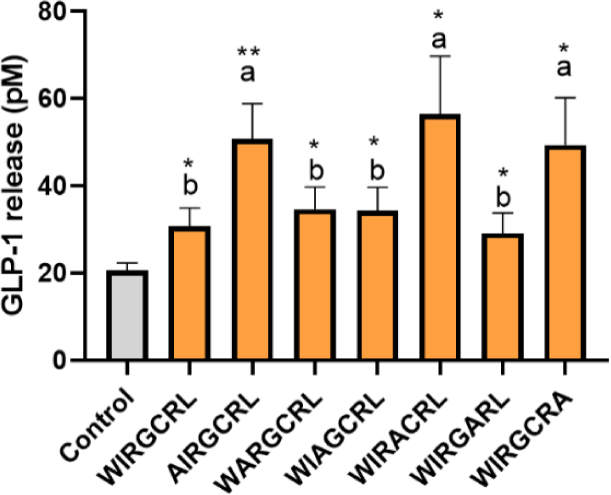
GLP-1 release after 1 h incubation of mouse intestinal organoids with 1 mM of synthetic peptides. Experiments were performed in triplicate, followed by technical duplicates. Error bars indicate SEM (*n* = 3). Statistical significance compared with control (one-way ANOVA with Tukey’s post hoc test) is indicated by *p < 0.05, **p < 0.01, ***p < 0.001, and ****p < 0.0001. Statistical significance (*p* < 0.05) in the comparison between different samples at the same dose is indicated by different letters (a, b).

There is little information about the structural features necessary to induce secretion by enteroendocrine cells of GLP-1. The organoid cellular media collected at 60 min were analyzed by HPLC–MS/MS in order to assess the possible proteolysis of the assayed peptides during the incubation. The small intestine brush border has different enzymes, oligopeptidases, lipases, and carbohydrases that contribute to nutrient hydrolysis and absorption.^27^ Other authors have described that enterocytes present in mouse intestinal organoids have mature brush borders, with the presence of their typical enzymes, including peptidases.^28^ Our results showed that, in all cases, the most abundant peptide corresponded to the assayed form, but the presence of shorter sequences in the supernatants highlights the action of proteolytic enzymes expected in organoids due to the differentiated brush border (Table S1). Concretely, the peptide ^123^WIRGCRL^129^ coexisted at 60 min with the shorter form, ^124^IRGCRL^129^. This hexapeptide was found to induce GLP-1 in STC-1, although at lower levels than the tryptophan-containing precursor peptide.^12^ Our results showed that the most susceptible sequences to the action of peptidases were WARGCRL, WIRACRL, and WIRGARL. The loss of N-terminal ^123^W was the most frequent cleavage, although the C-terminal loss of ^129^L was also detected, which reflects the activity of exopeptidases, the most abundant peptidases in the brush border membrane.^29^ Thus, although the STC-1 line would be the best model to perform extensive screenings to determine the hormonal release, the responses to food stimulants in cells and organoids are different. Organoids represent a more physiological model to study the GLP-1 signaling process, containing polarized cells, brush border enzymes, and expressing different types of receptors and transporters.

In order to investigate the pathways involved in the GLP-1 secretion in organoids, immunoblotting studies against phosphorylation of IRS1, AMPK, and ERK were performed (Figure S3). Our results in jejunal organoids showed that, after incubation with EWD, p-ERK was overexpressed approximately 2-fold at 15 and 30 min after sample exposure (Figure S3C), and probably, this phosphorylation was related to GLP-1 release. Previous studies in the mouse proximal colon observed an increase in p-ERK expression in response to stimulation with phenylalanine and tryptophan.^30^ In agreement with our results, the involvement of the MAP kinase pathway in GLP-1 secretion had been observed in enteroendocrine NCI-H716 after exposure to meat peptones.^31^ Although other authors had observed a negative relationship between AMPK phosphorylation and GLP-1 expression and secretion in STC-1 cells,^32^ no down expression of p-AMPK regarding to the control was detected at any of the analyzed time points with all assayed samples (Figure S3B).

Due to the low number of endocrine cells in organoids, the results of the pathways involved in the secretory effect of GLP-1 were only significant for p-ERK. The same experiment was performed in the STC-1 cell line. In this case, overexpression of p-ERK and down expression of p-AMPK after incubation with EWD at all time points was observed. The peptide induced an increase in ERK phosphorylation at 5 and 15 min and down expression of p-AMPK at 60 min, i.e., at the time points where the GLP-1 levels were significantly higher than the control (Figure 4D,C). No changes in p-IRS were observed in STC-1 cells after exposure with EWD or the peptide, although Phe induced a p-IRS overexpression at all time points (Figure S4B). Previous studies reported a decrease in the level of p-IRS when GLUTag cells were treated with a ketone body capable of inhibiting GLP-1 release. The authors suggested that this protein was involved in the activation of GPCRs through PI3K.^33^

**Figure 4:**
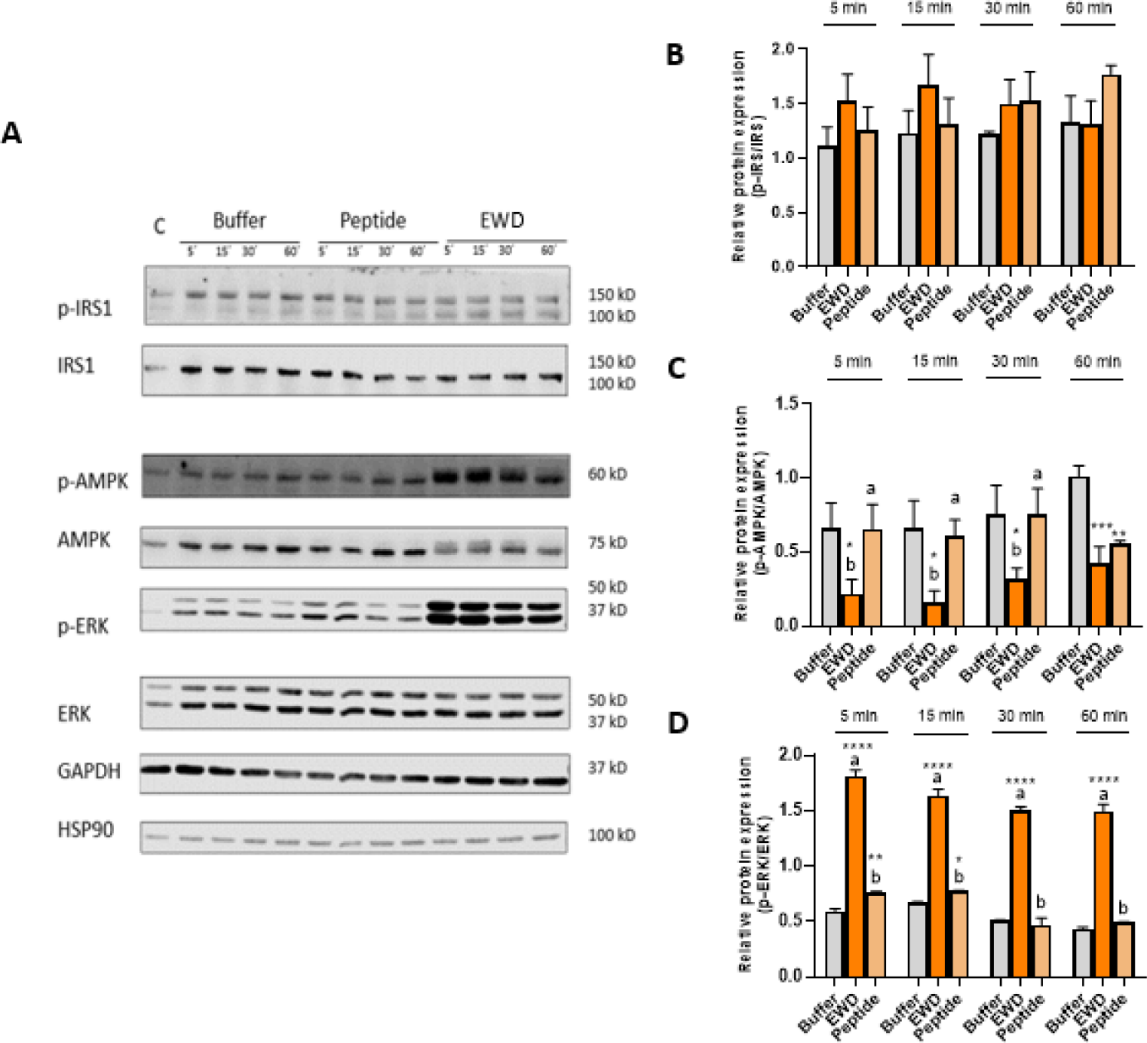
p-IRS1, IRS1, p-AMPK, AMPK, p-ERK, ERK, GAPDH, and HSP90 presence were analyzed at 5, 15, 30, and 60 min after peptide ^123^WIRGCRL^129^ (1 mM) or EWD (2 mg/mL) or buffer addition in STC-1 cells (A). Relative protein expression of p-IRS/IRS (B), p-AMPK/AMPK (C), and p-ERK/ERK (D). Error bars indicate SEM (*n* = 3). Statistical significance compared with control (one-way ANOVA with Tukey’s post hoc test) is indicated by *p < 0.05, **p < 0.01, ***p < 0.001, and ****p < 0.0001. Statistical significance (*p* < 0.05) in the comparison between different samples at the same time is indicated by different letters (a, b).

In summary, our results in organoids confirmed the phosphorylation of ERK after incubation with EW. In STC-1 cells, the phosphorylation of ERK and the down expression of p-AMPK in the presence of EWD were observed, while the peptide induced p-AMPK down expression.

### 3.3 Oral Glucose Tolerance Tests (OGTTs) in Wistar Rats and Incretin Levels in Plasma

Based on the GLP-1 results obtained in organoids and in line with the survival of ^123^WIRGCRL^129^ to simulated gastrointestinal digestion,^34^ OGTT was performed in Wistar rats. The effect of peptide ^123^WIRGCRL^129^ on blood glucose levels was compared with that of an equimolar mixture of the amino acids forming the peptide. The group receiving the peptide maintained glucose levels equal to basal levels until 15 min, while in the amino acid mixture-treated rats, glucose levels increased as in the control group receiving the glucose overload (Figure 5A). Our results evidence the ability of the peptide sequence to delay the maximum glucose peak by 30 min and ascribe this effect exclusively to the peptide sequence and not the individual amino acids. Several studies have demonstrated the hypoglycemic capacity of protein hydrolysates from different sources. For instance, a study showed that a pea hydrolysate, as well as the mixture of this product and metformin, can reduce blood glucose levels and improve glucose tolerance in diabetic mice, at doses used between 800 and 1600 mg/kg.^35^ Similarly, a camel milk hydrolysate showed hypoglycemic activity in an oral glucose tolerance test in diabetic rats when tested at 500 mg/kg.^36^ There are few studies with specific peptides, like peptide ^70^LPQNIPPL^77^ from Gouda cheese, which administrated at 300 mg/kg in rats was able to reduce blood glucose.^11^

**Figure 5:**
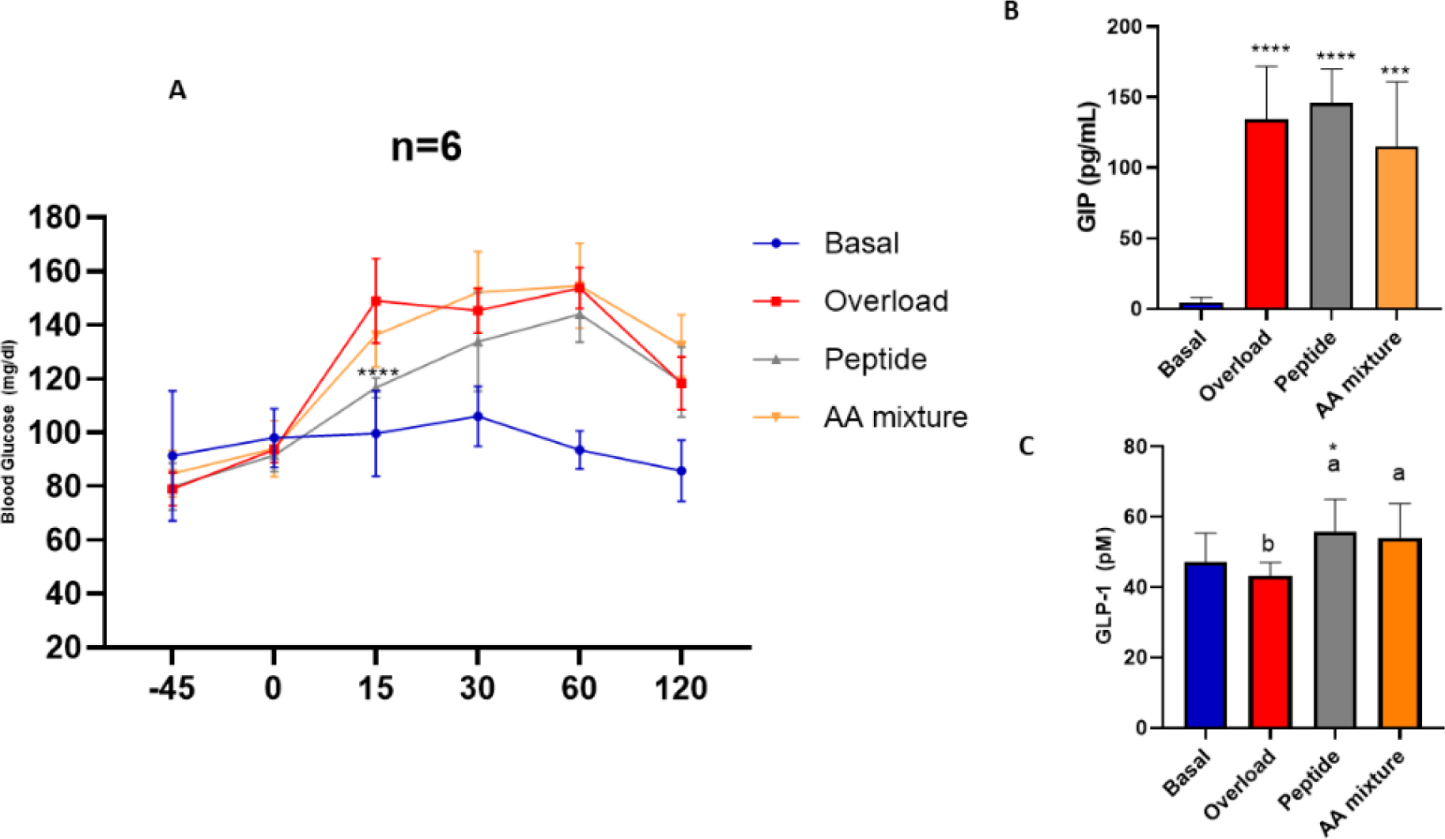
Blood glucose levels in mg/dL 45 min before the 3 mg/kg of glucose overload administration and after 15, 30, 60, and 120 min (A). GIP (B) and GLP-1 (C) plasma levels in mg/mL 15 min after glucose overload in basal, overload, peptide (^123^WIRGCRL^129^), and AA mixture groups. Statistical significance compared with control (one-way ANOVA with Tukey’s post hoc test) is indicated by *p < 0.05, **p < 0.01, ***p < 0.001, and ****p < 0.0001. Statistical significance (*p* < 0.05) in the comparison between different samples at the same dose is indicated by different letters (a, b).

In addition, incretin plasma levels 15 min after an oral glucose challenge were measured. Neither treatment with the peptide sequence nor with the amino acid mixture increased plasma GIP levels with regard to the overload glucose control group (Figure 5B). Significantly higher GLP-1 levels were found in rats pretreated with the peptide sequence (Figure 5C). This suggests that the delay in the maximum peak of glucose levels observed in the peptide group might be due to its GLP-1-inducing action, according to the potential GLP-1 secretory effect induced by the ^123^WIRGCRL^129^ sequence in STC-1 cells and mouse jejunal organoids.

In summary, further studies are needed to establish the link between the action of these and other peptides released during digestion and the release of endogenous GLP-1 stores. However, our results show that the GLP-1 response in a model that preserves the main phenotypic features and functional characteristics of the intestine, such as mouse jejunal organoids, is slightly different from that found in STC-1 cells, especially for free amino acids like Phe. This underscores the importance of using both STC-1 cells and organoids to gain a more comprehensive understanding of the mechanisms underlying GLP-1 secretion, while our findings also highlight the potential of egg white peptides as an alternative to conventional treatments for obesity and type 2 diabetes due to their strong GLP-1-inducing capacity.

## Supplementary Material



## Notes

Relative *CCK* and *PYY* gene expression in mouse intestinal organoids (Figure S1); relative *CCK* and *PYY* gene expression in STC-1 cells (Figure S2); percentage of each sequence found by HPLC/MS-MS in organoids after incubated of synthetic peptides (Table S1); Western blot in organoids in the presence of peptide and EWD (Figure S3); Western blot in STC-1 cells in the presence of Phe (Figure S4) (PDF)

## Abbreviations

GLP-1: glucagon-like peptide-1
Phe: phenylalanine
ERK: extracellular signal-regulated kinase
AMPK: AMP-activated protein kinase
EWD: lyophilized supernatant after the digestion of egg white following the INFOGEST protocol
PEPT-1: peptide transporter 1
SGLT: sodium-coupled glucose transporter
A: alanine
ISC: intestinal stem cell
BSA: bovine serum albumin
DMEM/F12: Dulbecco’s Modified Eagle’s Medium/Ham’s Nutrient Mixture F12
DMEM: Dulbecco’s Modified Eagle’s Medium
qPCR: real-time PCR
CCK: cholecystokinin
PSA: penicillin–streptomycin–amphotericin
PYY: peptide YY
HPLC–MS/MS: high-performance liquid chromatography–tandem mass spectrometry
GAPDH: glyceraldehyde-3-phosphate dehydrogenase
HSP90: heat shock protein
OGTT: oral glucose tolerance test
GIP: gastric inhibitory polypeptide
R: arginine
W: tryptophan
G: glycine
L: leucine
Y: tyrosine
