# Supplementary material for: A Lysozyme-Derived Peptide Induces GLP‑1 Secretion in Mouse Jejunal Organoids and Modulates Glycemia in Rats
Source: J Agric Food Chem. 2025 Jun 18;73(26):16420–8. doi: 10.1021/acs.jafc.5c05148 (PMC12232376; doi:10.1021/acs.jafc.5c05148)
Supplement: Supplementary file 1 [file jf5c05148_si_001.pdf]

Supplementary Material

A lysozyme-derived peptide induces GLP-1 secretion in mice jejunal organoids and modulates glycemia in rats

Santiaga María Vivanco-Maroto[1], María-Carmen López de las Hazas[2], Esperanza Herradón[3,4], Rocío Girón[3,4], Beatriz Miralles[1] Nancy Paniagua[3,4], Visitación López-Miranda[3,4], Alberto Dávalos[2,5], Isidra Recio[1,*]

[1] Instituto de Investigación en Ciencias de la Alimentación, CIAL (CSIC-UAM, CEI UAM+CSIC), Nicolás Cabrera, 9, 28049 Madrid, Spain.

[2] Laboratory of Epigenetics of Lipid Metabolism, Madrid Institute for Advanced Studies Food (IMDEA Food), CEI UAM + CSIC, Carretera de Canto Blanco, 8, 28049 Madrid, Spain

[3] Área de Farmacología, Nutrición y Bromatología, Dpto. C.C. Básicas de la Salud, Facultad de Ciencias de la Salud, Universidad Rey Juan Carlos, Unidad Asociada I+D+i al Instituto de Química Médica (CSIC), 28922 Alcorcón, Spain

[4] High Performance Research Group in Experimental Pharmacology (PHARMAKOM), Spain

[5] Consorcio CIBER de la Fisiopatología de la Obesidad y Nutrición (CIBERObn), Instituto de Salud Carlos III (ISCIII), Madrid, Spain.

* Corresponding author: I. Recio

Nicolás Cabrera, 9. 28049 Madrid, Spain

Phone: +34 910017940 13

Fax: +34 910017905 14

e-mail: i.recio@csic.es

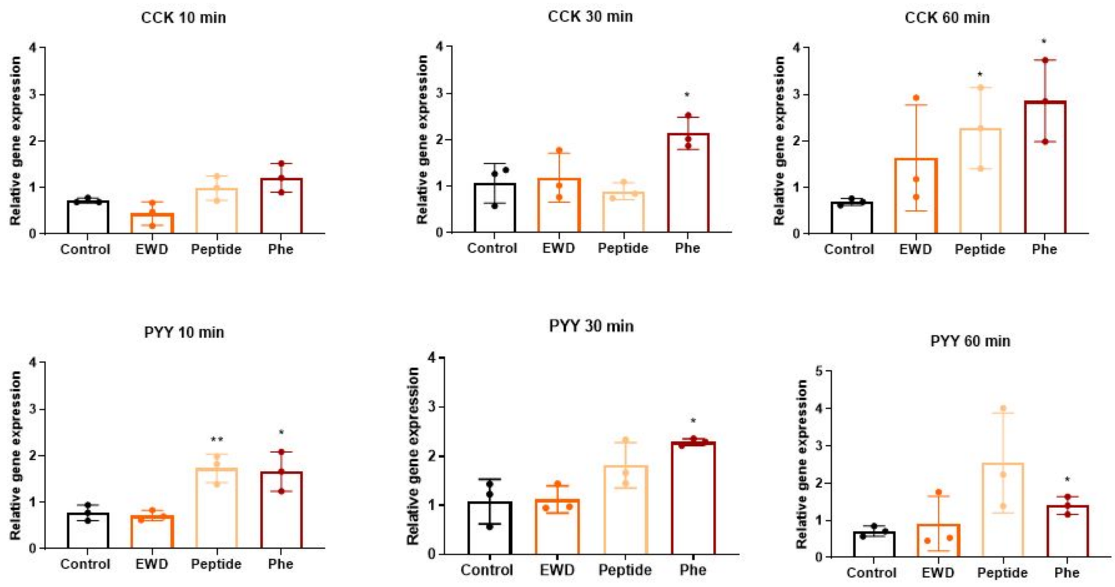

Figure S1. Relative CCK and PYY gene expression in mice intestinal organoids 10, 30 or 60 min after EWD (2 mg/mL), ^{123}WIRGCRL^{129} (peptide, 1 mM), Phe (20 mM) or buffer (control) given as relative expression (fold change) versus the control. Experiments were performed in triplicate, followed by technical duplicates. Error bars indicate SEM (n = 3). Statistical significance compared with control (unpaired t-test) is indicated by $^{*}p < 0.05$, $^{**}p < 0.01$, $^{***}p < 0.001$ and $^{****}p < 0.0001$.

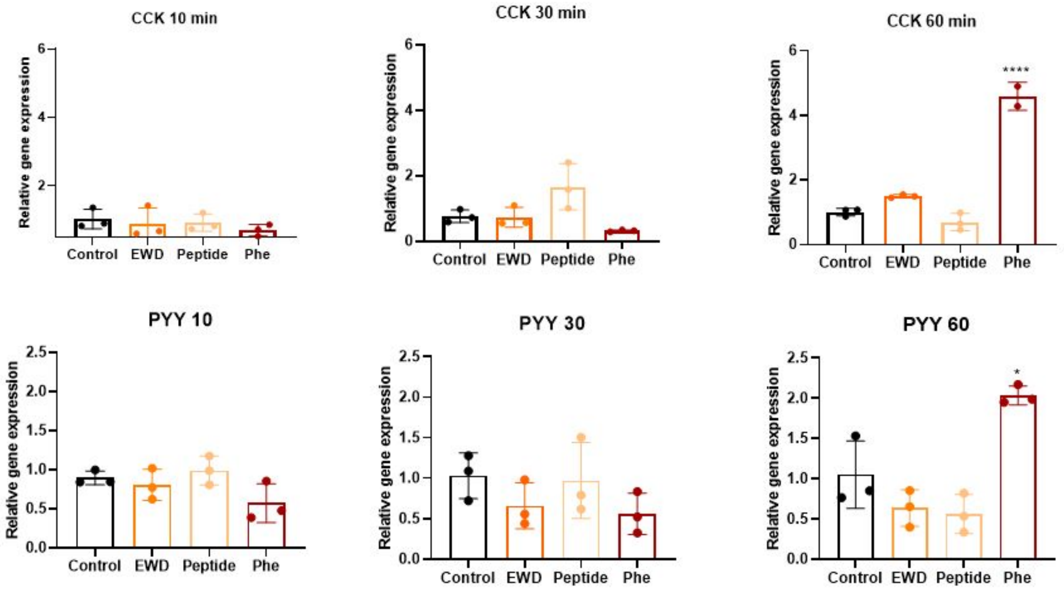

Figure S2. Relative *CCK* and *PYY* gene expression in STC-1 cells 10, 30 or 60 min after EWD (2mg/mL), ^{123}WIRGCRL^{129} (peptide, 1 mM), Phe (20 mM) or buffer (control) addition. Experiments were performed in triplicate, followed by technical duplicates. Error bars indicate SEM (n = 3). Statistical significance compared with control (unpaired t-test) is indicated by $^{*}p < 0.05$, $^{**}p < 0.01$, $^{***}p < 0.001$ and $^{****}p < 0.0001$.

Table S1. Peptide forms and percentage of peptide sequences identified by HPLC/MS-MS in organoids supernatants 1 hour after the incubation with the synthetic peptides (1 mM).

Assayed sequence	Peptide forms identified	%
WIRGCRL	IRGCRL	5.84
	WIRGCRL	94.16
AIRGCRL	IRGCRL	6.30
	AIRGCRL	93.70
WARGCRL	ARGCRL	11.54
	WARGCR	1.14
	WARGCRL	87.33
WIAGCRL	IAGCRL	3.61
	WIAGCRL	96.36
WIRACRL	IRACRL	12.46
	WIRACRL	88.92
WIRGARL	IRGARL	28.47
	WIRGARL	71.53
WIRGCRA	IRGCRA	3.27.
	WIRGCR	3.59
	WIRGCRA	93.14

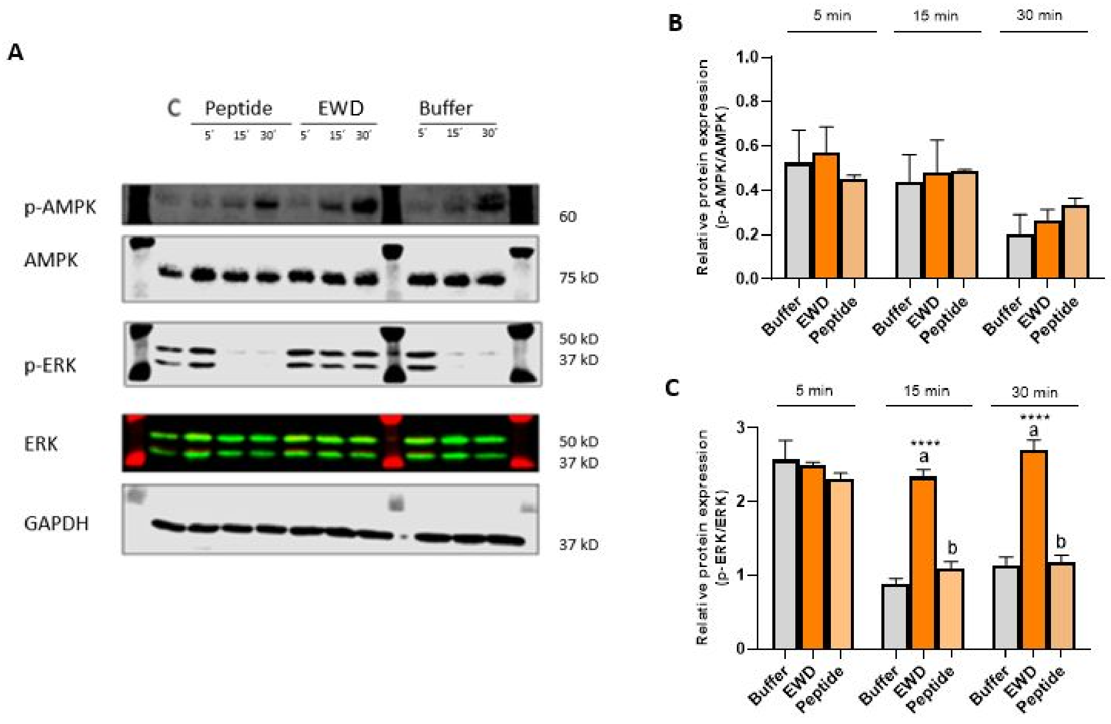

Figure S3. p-IRS1, IRS1, p-AMPK, AMPK, p-ERK, ERK, GADPH, and HSP90 was analyzed 5, 15, 30 and 60 min after [123]WIRGCRL[129] (1 mM) or EWD (2 mg/mL) or buffer addition in organoids (A). Relative protein expression of p-AMPK/AMPK (B) and p-ERK/ERK (C). Error bars indicate SEM (n = 3). Statistical significance compared with control (one-way ANOVA with Tuke´s post hoc test) is indicated by *$p < 0.05$, **$p < 0.01$, ***$p < 0.001$ and ****$p < 0.0001$. Statistical significance ($p < 0.05$) in the comparison between different samples at the same time is indicated by different letters (a, b).

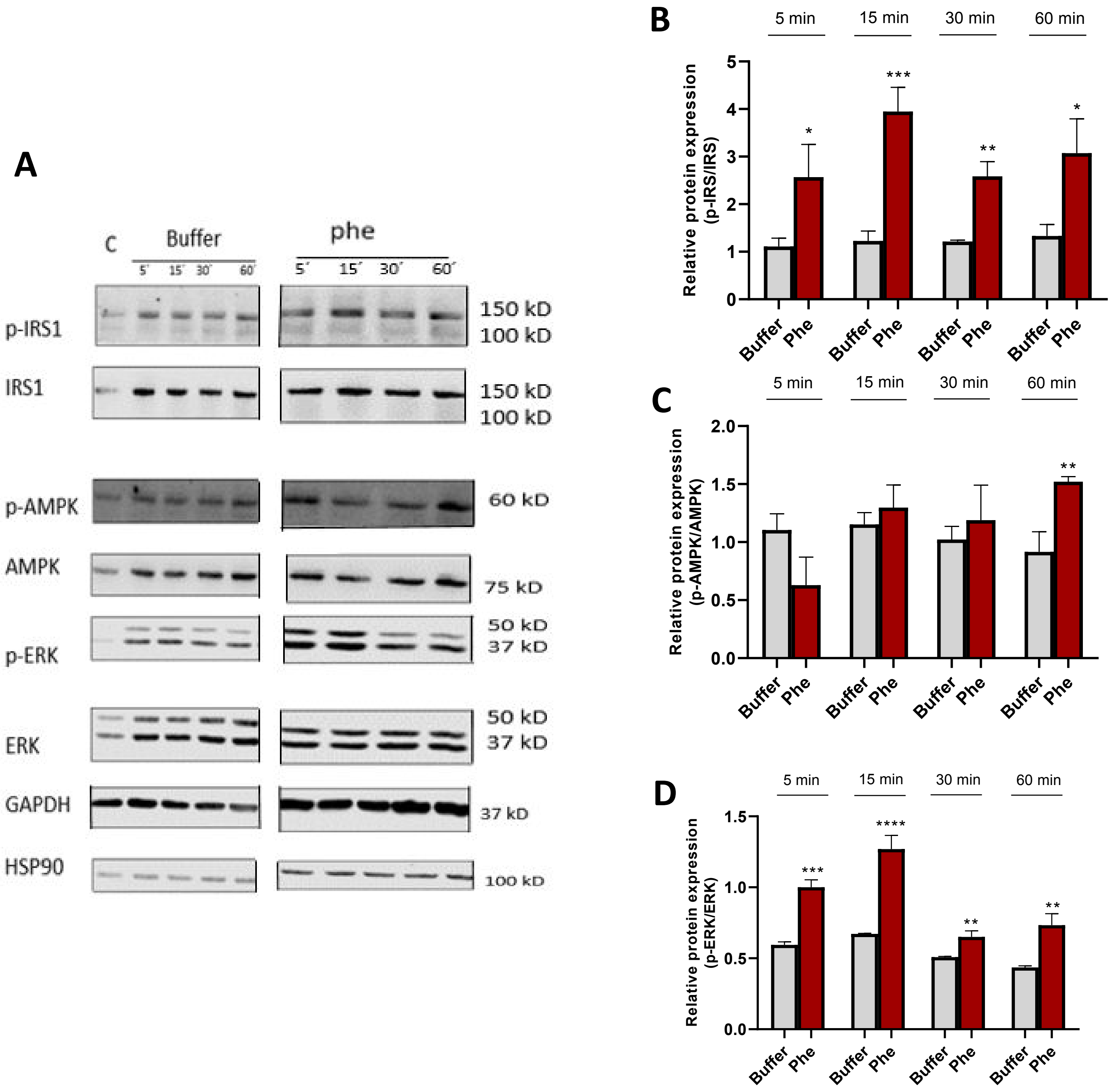

Figure S4. A. p-IRS1IRS1, p-AMPKAMPK, p-ERK, ERK, GADPH, and HSP90 presence was analyzed 5, 15, 30 and 60 min after Phe (20 mM) or buffer addition in STC-1 cells (A). Relative protein expression of p-IRS/IRS (B), p-AMPK/AMPK (C) and p-ERK/ERK (D). Error bars indicate SEM (n = 3). Error bars indicate SEM (n = 3). Statistical significance compared with control (unpaired t-test) is indicated by *p < 0.05, **p < 0.01, ***p < 0.001 and ****p < 0.0001.
